# The Past Is Never Dead—Measles Epidemic, Boston, Massachusetts, 1713

**DOI:** 10.3201/eid2107.150397

**Published:** 2015-07

**Authors:** David M. Morens

**Affiliations:** National Institutes of Health, Bethesda, Maryland, USA

**Keywords:** measles, epidemic, Boston, Massachusetts, viruses, Cotton Mather

The recent measles epidemic in the United States has aroused public disbelief that a disease well-controlled for decades is reemerging to threaten children in the United States. Controversy surrounds measles vaccination in the United States; some parents have even avoided vaccinating their healthy children by exposing them to measles-infected children. However, measles has repeatedly reemerged in the United States during the past 3 centuries or longer (*1*,*2*), and its emergence patterns and means of preventing and controlling it are well understood. Until measles is globally eradicated—a goal within reach—it will continue to reappear, sicken, and kill almost anywhere, and we must energetically control each outbreak.

When we consider modern measles prevention, it is worth recalling what epidemics were like before vaccines and organized public health systems. One vivid account of measles describes the disease’s deadly spread through a prominent Boston household >300 years ago. In 1713, America’s first important medical figure (*3*), Puritan minister Cotton Mather (1663–1728), called by one authority “the Dr. Spock of the colonial New England” (*4*), wrote about a measles epidemic in the American colonies, describing not only its epidemiology and devastation but also the fear it elicited. Mather’s account reminds us of the need for such modern medical and public health tools as vaccination, patient isolation, and prevention policies in saving families from the once-unpreventable diseases that compelled us to develop effective medical advances in the first place.

The following account, condensed from Cotton Mather’s personal diary (*5*), focuses on illnesses in his own household, including those of his wife, 9 children, and a maidservant, over the course of 6 weeks during October–November, 1713.

## Diary Excerpts

[18 October] …The Measles coming into the Town, it is likely to be a Time of Sickness…

[19 October] [I must]… lay hold on the Occasion to awaken Piety, and Preparation for Death, in the Souls of the children.

[24 October]… [on ≈18 October] my Son *Increase* fell sick…

[26 October] I must quicken the preparation of my Domesticks…

[27 October] My desirable Daughter *Nibby*, is now lying very sick of the *Measles…*

[28 October] … a very sensible Calamity is begun upon the Town… [with] some Degree of Mortality.

[30 October] The Spreading of the Measles… [is much worse in] Families, where they conflict with Poverty… This day, my Consort [wife], for whom I was in much Distress, lest she should be arrested with the Measles which have proved fatal to Women that were with child, after too diligent an Attendance on her sick Family, was… surprized with her Travail [went into labor]… [and] graciously delivered her, of both a Son and a Daughter… wherein I receive numberless Favors of God. My dear *Katy*, is now also down with the *Measles*…

[1 November] *Lord’s Day*. This Day, I baptized my new-born twins… So I called them, *ELEAZAR* and *MARTHA*….

[4 November] In my poor Family, now, first, my Wife has the *Measles* appearing on her…

My Daughter *Nancy* is also full of them…

My Daughter *Lizzy*, is likewise full of them…

My Daughter *Jerusha*, droops and seems to have them appearing.

My Servant-maid, lies very full and ill of them.

Help Lord; and look mercifully on my poor, sad, sinful Family…

[5 November] My little son *Samuel* is now full of the Measles….

[7 November]… my Consort is in a dangerous Condition, and can gett no rest... Death… is much feared for her… So, I humbled myself before the Lord, for my own Sins... that His wrath may be turned away…

[8 November] …For these many Months… I have often, often express’d my Fear unto my Friends concerning [the measles]. And now, *the Thing that I greatly feared is coming upon me!*

*…*this Day we are astonished, at the surprising Symptomes of death upon [my wife]… Oh! The sad Cup, which my Father has appointed me!... God made her willing to Dy. God extinguished in her the Fear of Death… God enabled her to Committ herself into the Hands of a great and good Savior; yea, and to cast her Orphans there too…

I pray’d with her many Times, and left nothing undone…

[9 November] On Munday… [9 November], between three and four in the Afternoon, my dear, dear, dear Friend expired…. [I] cried to Heaven…

[10 November] …I am grievously tried, with the threatening Sickness of my discreet, pious, lovely Daughter *Katharin.*

And a Feavour which gives a violent Shock to the very Life of my dear pretty *Jerusha*.

[11 November] This day, I interr’d the earthly part of my dear consort…

[12 November] …The epidemical Malady began upon this Town, is like to pass thro’ the Countrey…. it [might] be a service unto the public, to insert in the News-paper, a brief Direction for the managing of the sick. I will advise with a Physician or two.

[13 November] …I hear of some aged and bedrid people, which I design speedily to visit…

[14 November] This Morning… the death of my Maid-servant, whose Measles passed into a malignant Feaver…

Oh! The trial, which I am this Day called unto in… the dying Circumstances of my dear little *Jerusha*!

The two Newborns, are languishing in the Arms of Death…

[15 November] … my little *Jerusha*. The dear little Creature lies in dying Circumstances. Tho’ I pray and cry to the Lord… Lord she is thine! Thy will be done!...

[18 November] …About Midnight, little *Eleazar* died.

[20 November] …The distressed Families of the Poor to which I dispense… are now so many…

Little *Martha* died, about ten a clock, A.M.

I begg’d, I begg’d, that such a bitter Cup, as the Death of that lovely [Jerusha], might pass from me…

[21 November] …Betwixt 9 h. and 10 h. at night, my lovely *Jerusha* Expired. She was 2 years, and about 7 months old. Just before she died, she asked me to pray with her; which I did… and I gave her up unto the Lord. [Just as she died] she said, *That she would go to Jesus Christ*…

Lord I am oppressed; undertake for me!

[23 November] …My poor Family is now left without any Infant in it, or any under seven Years of Age…

This day, I followed my dear *Jerusha* to the Grave... with Resolutions… especially what I may do for my own and other Children.

[25 November] … several Things may I do for the Service of the Town in its Adversity… [including] Charitable Distributions among the Poor… I will procure, and I will dispense, as many of these, as I can…

[28 November] Breathing in the midst of so many Deaths, what can there be so needful and so proper for me, as for me to *Die Daily*, and become a man dead unto this World…

[17 December] This day was kept as a Day of Prayer in the several Churches of Boston, because of the heavy Calamity on the Town. And a liberal Collection was made, for the Relief of the Poor, under the Calamity of Sickness, and growing Scarcity. It was a most bitter season…

[23 December] …I have given to the Printer, a Letter about the *Right Management of the Sick under the Distemper of the Measles* [note: the actual publication bears a different title; see Mather, *6*]; which is now spreading and raging in the Countrey. I propose to scatter it into all parts… to save many lives….

## Discussion

Mather’s chronicle of explosive measles in his own family documents a shocking case-fatality rate (5 of 11 infected household members, or 45%). It also reminds us that emerging and reemerging diseases such as measles once appeared suddenly to kill almost anyone (*7*), a reality with which most persons lived until modern times (and with which many in the developing world who lack access to good nutrition and modern medical care still live today).

The fear that measles engendered in the citizens of Boston, expressed in Mather’s many diary entries of tenderness, hope, and despair, should bring to mind another disease of similar case-fatality that arouses comparable fear and despair: Ebola virus disease. The ravages of this disease in West Africa have recently been broadcast on television screens in the United States. Like the stunned looks in the eyes of grieving West Africans, the account of a father watching his family suffer and succumb to death elicits sympathy for those struggling against fatal diseases that even modern parents may not fully understand, and that even aggressive public health measures may prevent only with great difficulty. The writer of these passages was not just America’s preeminent theologian and medical authority, but a husband and father whose grief can be universally understood. One of Mather’s most noteworthy sermons, preached 24 years earlier, observed that: “Yet few outward Earthly Anguishes are equal unto these. The Dying of a Child is like the Tearing off [of] a limb…” (*8*).

Several medical aspects of Mather’s entries are noteworthy. From Mather’s brief notations, we cannot be certain of the exact dates of onset of most of the illnesses he described. However, he clearly chronicles 2 serial generations of measles within his family (one of no more than 17 days, the other of no more than 15). These intervals correspond to what textbooks began to describe, more than a century later, about the patterns of measles spread. Measles seems to have been brought into the household by Mather’s son Increase around October 18, 1713. Increase apparently infected his mother, Elizabeth Clark-Hubbard Mather, and his 4 youngest siblings; these 5 became ill on or shortly before November 4. A second generation of measles involved twins, born on October 30 and said to be ill on November 14, consistent with infection at or shortly after birth (or, much less likely, given the apparent date of onset of disease in their mother, infected in utero)*.* It is curious that Mather correctly understood measles to be especially dangerous for parturient women. He may have concluded this on the basis of reports circulating among physicians or because he knew that the same phenomenon had long been documented for influenza; an influenza pandemic complicated by pleuropneumonia had begun, and swept through the Americas, during 1697–1698.

Also noteworthy, and mentioned in his referenced letter (*6*) but not specifically in the diaries, is Mather’s realization that pleuritick fever, probably corresponding to pneumonia, was a serious complication of measles. Viral pneumonia and secondary postmeasles bacterial pneumonia are now considered to be among the most fatal complications of this disease (*9*,*10*).

Today, high case-fatality rates for measles are seen only in ill and immunosuppressed persons or in those who are malnourished. However, in the premodern world measles was, confusingly, sometimes benign, sometimes deadly. The reasons for this documented pattern remain obscure: differences in virulence among the various extant measles clades have not been found. The deadliest historical measles outbreaks seemed to occur disproportionately in the poor and disadvantaged, especially including young children in orphanages or environments of desperate poverty, or in indigenous persons living in areas of potential relative nutritional deficiency (*9*). These findings suggest a key role for host and environmental factors in measles severity. Even a modest deficiency in vitamin A is now known to exacerbate measles severity, and postmeasles bacterial pneumonias appear to be much more common in situations of poverty, crowding, and high bacterial circulation.

The *Letter* Mather promised was “published for the benefit of the poor” (*6*) in December 1713. It informed those unfortunate citizens without access to a physician’s care about the typical clinical appearance and course of measles, and about simple treatments for it. In recommending generic remedies for unbalanced “humours,” it broke no new ground, suggesting (italics and capitalization preserved): *Syrup of Saffron and Treacle Water*, Syrup(s) of Maiden-hair or *Hyssop*, Tea of *Sage* or *Rosemary, Sugar-Candied*, or *Buttered Pills*, *Hot Beer and Rum*, *Hot Cyder*, *Hot Honey*, *Water with Roasted Apples* in it, Shavings of *Castile Soap* in a Glass of Wine or Beer, or *Tea* made of *Rhubarb*, and sweetened with a Syrup of *Marshmallow* (*Althaea officinalis*). These were all ingredients that the poor could afford, and that might at least be comforting, if not life-saving.

The 1713 Boston measles epidemic occurred 21 years after the Salem Witch Trials, in which historians still debate Mather’s role as instigator or mitigator; 7 years after Mather discovered that inoculation might be able to prevent smallpox; and 8 years before Mather passionately advocated inoculations in response to a deadly smallpox epidemic. Because Mather died 30 years before preventive measles inoculation is known to have been attempted (*11*) and 225 years before the first effective measles vaccine was developed, we have no way of knowing what he would have thought about measles immunogens, their use in public health programs, or policies to ensure universal vaccination of children.

In this writer’s opinion, however, there is little doubt that Mather—were he alive today—would strongly support all reasonable measles control efforts, including universal and publicly enforced vaccination. After all, he was a proponent of smallpox inoculation, and he fought energetically in public forums against all who tried to prevent inoculations on the grounds that it was inherently risky and might theoretically prolong or even start epidemics. He also had lived through the most devastating tragedy of his life: the loss of his own wife and children from epidemic measles. Moreover, as the first person in the New World to espouse an “animalcular” theory (germ theory) of disease (*3*), Mather would surely have been predisposed to accept the scientific basis of immunization, and he surely would have been impressed that aggressive global measles vaccination has, in little more than a decade, reduced the death rate by a factor of at least 5-fold and saved ≈1 million lives each year. It seems highly likely that Mather would not only advocate measles prevention and control, on the basis of the most up-to-date medical tools and public health information, but also measles elimination and eradication.

Mather’s grief and despair, expressed in line after line of his diary, should remind us not only that the risks of infectious diseases like measles are real and ongoing but also why previous generations of physicians and scientists, supported by a public desperate for medical advances to save their children, worked so long and so well to develop and deploy the very vaccines that some people now avoid and decry. As we debate today how best to deal with yet another measles epidemic in the United States, we should look closely at the lessons Cotton Mather and his contemporaries learned 3 centuries ago. Emerging infectious diseases like measles keep reminding us that “the past is never dead. It isn’t even past” (*12*).
